# Activation of superior colliculi in humans during visual exploration

**DOI:** 10.1186/1471-2202-8-66

**Published:** 2007-08-14

**Authors:** Marc Himmelbach, Michael Erb, Hans-Otto Karnath

**Affiliations:** 1 Section Neuropsychology, Center for Neurology, Hertie-Institute for Clinical Brain Research, Eberhard Karls University, Hoppe-Seyler-Str. 3, 72076 Tübingen, Germany; 2 Section for Experimental NMR, Department of Neuroradiology, Eberhard Karls University, Hoppe-Seyler-Str. 3, 72076 Tübingen, Germany

## Abstract

**Background:**

Visual, oculomotor, and – recently – cognitive functions of the superior colliculi (SC) have been documented in detail in non-human primates in the past. Evidence for corresponding functions of the SC in humans is still rare. We examined activity changes in the human tectum and the lateral geniculate nuclei (LGN) in a visual search task using functional magnetic resonance imaging (fMRI) and anatomically defined regions of interest (ROI). Healthy subjects conducted a free visual search task and two voluntary eye movement tasks with and without irrelevant visual distracters. Blood oxygen level dependent (BOLD) signals in the SC were compared to activity in the inferior colliculi (IC) and LGN.

**Results:**

Neural activity increased during free exploration only in the SC in comparison to both control tasks. Saccade frequency did not exert a significant effect on BOLD signal changes. No corresponding differences between experimental tasks were found in the IC or the LGN. However, while the IC revealed no signal increase from the baseline, BOLD signal changes at the LGN were consistently positive in all experimental conditions.

**Conclusion:**

Our data demonstrate the involvement of the SC in a visual search task. In contrast to the results of previous studies, signal changes could not be seen to be driven by either visual stimulation or oculomotor control on their own. Further, we can exclude the influence of any nearby neural structures (e.g. pulvinar, tegmentum) or of typical artefacts at the brainstem on the observed signal changes at the SC. Corresponding to findings in non-human primates, our data support a dependency of SC activity on functions beyond oculomotor control and visual processing.

## Background

A vast number of electrophysiological and lesion studies in animals demonstrated the involvement of the SC in oculomotor control [1-3]. Evidence for corresponding functions of the SC in humans is still rare in comparison to our knowledge of cortical components of the human oculomotor and visual system. A small number of neuropsychological case studies in humans with isolated damage to the SC reported deficient oculomotor behaviour and attentional deficits [4-6]. Likewise, activity of the superior tectum was specifically investigated only in a small number of fMRI studies focusing on visual processing [7-9], audio-visual integration [10], and (oculo)motor control [11,12]. The scarcity of neuroimaging studies explicitly investigating the function of the SC in humans might be due to methodological drawbacks associated with fMRI measurements of the tectum. The small volume of the SC and motion-related artefacts caused by the blood-flow in nearby large vessels [13,14] hamper functional measurements of the tectum due to increased physiological noise in comparison to cortical areas and other subcortical structures like basal ganglia and thalamus. While such noise could hinder the detection of true signals, it could also cause (task-related) artefacts that might be misinterpreted as collicular activation. Therefore, some studies employed sophisticated methods like cardiac-triggered data acquisition [7] or incorporating regressors for cardiac-cycle related noise in statistical models [9].

Nevertheless, hitherto published studies already added a great deal of knowledge about the human SC. Not only has the contralateral representation of visual input in the right and left SC been demonstrated [7,8], but also the medio-lateral organisation of upper and lower visual field stimulation [8]. Just recently, a high-resolution fMRI analysis demonstrated greater BOLD signals for monocular visual stimuli presented in the temporal hemifield than for stimuli presented in the nasal hemifield under monocular viewing conditions [9]. Surprisingly, only very few studies explicitly reported oculomotor activations of the SC. In fact, a quite recent general survey of fMRI studies on the oculomotor system included no section on the functional involvement of the SC in oculomotor control at all [15]. This is quite surprising given that a vast amount of non-human research demonstrated the oculomotor functions of the SC [1-3]. Using a slow event-related fMRI design, Petit and Beauchamp [12] investigated activations correlated with the execution of eye-, head-, and gaze-movements. This design allowed for the differentiation between the immediate onset of motion artefacts and delayed physiological BOLD-responses. They found an equivalent involvement of the SC in all three types of movements [12]. This result was in agreement with electrophysiological data from non-human species demonstrating a dependency of SC activity not on saccade end points but on more complex behaviour and behavioural goals. SC neurons seem to encode gaze direction implemented by combined head and eye movements [16,17] and demonstrate continuous, goal-related activity changes during multi-step saccades [18-20]. Some studies even revealed activity changes associated with cognitive operations beyond sensorimotor integration, i.e. encoding of the behavioural relevance of stimuli [21,22] and selection of targets among distracters during visual search [23,24]. The latter, visual search has been investigated in humans using fMRI several times. Only one study, however, explicitly reported activation of the SC [11]. The authors found significantly higher signals during visual exploration in comparison with saccades to two horizontal targets in a cluster partly enclosing the SC. However, prior to statistical analysis, functional volumes were heavily smoothed. Therefore, in all probability surrounding areas, e.g. dorsal tegmentum and posterior thalamus (pulvinar), have also contributed to the analysed signal behaviour of local maxima in the SC. Signal differences between conditions could even be (partly) driven by systematic artefacts due to typical physiological noise at the brain stem [13,14]. Furthermore, the comparisons conducted only between visual exploration and saccades to horizontal targets, on the one hand, and only between visual exploration and visual stimulation (without the execution of eye-movements), on the other hand, could not control for the combined effects of saccades and visual input. Consequently, their analysis of effective connectivity between the SC and cortical areas also revealed confounded effects: They observed relevant input not only from oculomotor but also from primary visual cortex [11].

**Figure 1:**
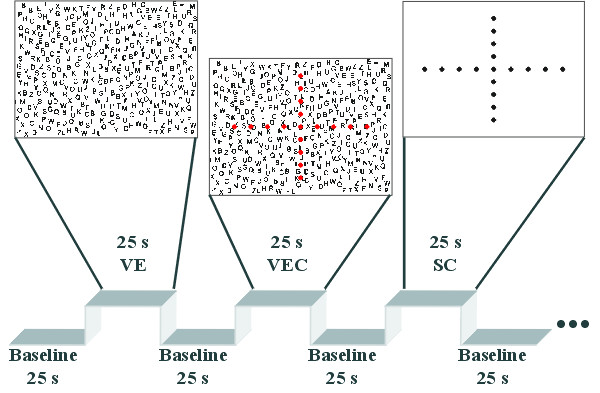
**Experimental conditions**. An initial baseline period of fixation was followed by visual exploration (VE). A letter array of 24 × 35 cm^2 ^(~9° × 13°) was presented consisting of about 370 letters set in Arial font with a vertical size of 0.95 cm (~0.36°) per letter at a viewing distance of 150 cm. In the saccade block with visual background (VEC), a cross of red circles was presented in front of the same letter field. In the saccade block without visual background (SC), the same cross was presented, but without the letter field. Each line consisted of 9 dots of a size of 0.5 cm (0.2°) at a distance of 2.75 cm (1°) from each other. A cycle consisting of these three conditions interleaved with baseline periods was repeated 5 times during each of the three experimental runs.

Recently, we studied activity changes during a free visual exploration task in humans using fMRI [25]. In contrast to the study by Gitelman et al. [11], free visual exploration of a crowded stimulus display was now compared to (i) the execution of voluntary eye movements to predetermined target positions in front of the same visual stimulus display and (ii) the execution of voluntary eye movements to target positions without further visual stimuli (Figure 1). The whole brain comparison between visual search and saccade execution without additional stimuli revealed a cluster partly covering the SC as well as other subcortical areas. However, like the previous study by Gitelman et al. [11], our analysis was based on smoothed functional data which created the same caveats in ascribing these signal changes to the SC.

**Figure 2:**
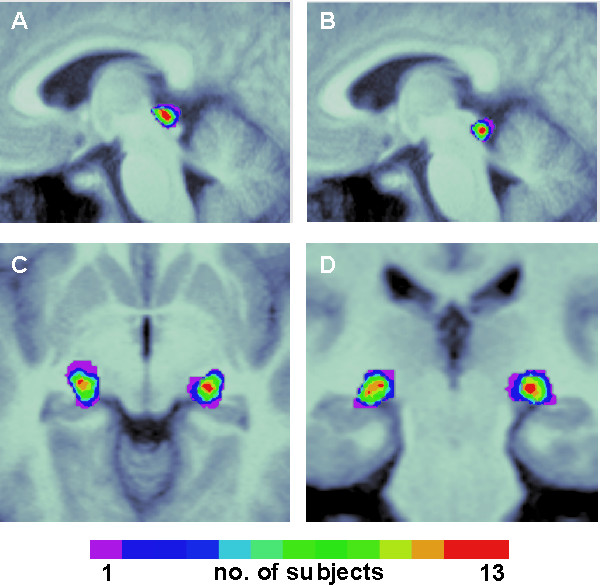
**Anatomical regions of interest**. Overlap of superior (A) and inferior (B) colliculus ROIs of all subjects illustrated on a sagittal slice of the group's mean brain (x = 4 mm). (C, D) Overlap of LGN ROIs illustrated on a transversal (C, z = -6) and coronal (D, y = -25) slice of the group's mean brain. The number of overlapping ROIs is colour coded, from violet (n = 1) to red (n = 13).

Therefore, we conducted a re-analysis of our data refraining from smoothing before statistical analysis. We determined anatomical ROIs individually in every subject (Figure 2) and conducted an analysis of regional signal changes. Our aim in doing so was to clarify whether consistent activity changes associated with visual exploration could be attributed to the SC and also whether they depended on exploratory activity, the execution of saccades, or on visual input. To control for unspecific global signal changes and/or localised artefacts at the dorsal brainstem, we contrasted data from the superior colliculus with that from the inferior colliculus during the tasks. The latter are well known not to be involved in visually guided eye movement tasks and thus should not show any task-related signal changes. Further, we analysed signal changes at the LGN in order to demonstrate increased activation associated with visual stimulation.

## Results

The subjects executed a higher number of saccades during visual exploration in comparison to both voluntary saccade tasks. This difference could not have been avoided a priori as the paradigm was based on the execution of voluntary eye movements that could not be triggered (for a detailed discussion, please see [25]). We controlled for this variable by including the individual saccade frequency for each subject in each condition as a covariate in a multivariate analysis of variance (MANOVA) of signal changes in the superior and inferior colliculi and LGN.

**Figure 3:**
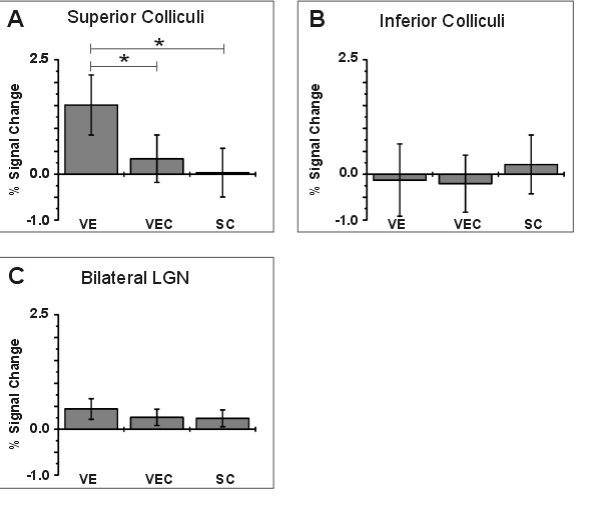
**BOLD signal changes**. Estimated marginal means of BOLD signal changes across all subjects with 95% confidence interval for each experimental condition in the superior colliculi (A), inferior colliculi (B), and lateral geniculate nuclei (C).

While the multivariate test for the effect of experimental conditions on signal changes in superior colliculi, inferior colliculi, and bilateral LGN failed statistical significance (F_6,68 _= 1.616, p = 0.156), subsequent univariate statistics revealed a significant effect of the experimental condition on the signal behaviour in the superior colliculi (F_2,35 _= 4.819, p = 0.014). In contrast, the differences between the experimental conditions in the inferior colliculi and bilateral LGN were far from being significant (IC: F_2,35 _= 0.571, p = 0.570; LGN: F_2,35 _= 0.821, p = 0.448). Pairwise comparisons of the estimated marginal means of the experimental conditions for the superior colliculi revealed significant differences after Bonferroni-correction for multiple comparisons between free visual exploration and saccades with irrelevant stimuli (VE: 1.511, 95% Confidence interval (CI): 0.855 – 2.166; VEC: 0.340, CI: -0.177 – 0.858; p = 0.049) and between free visual exploration and saccades without irrelevant stimuli (SC: 0.037, CI: -0.496 – 0.571; p = 0.012) (Figure 3a). The analysis revealed no significant effect of the covariate saccade frequency on any of the structures investigated here (SC: F_1,35 _= 2.506, p = 0.122; IC: F_1,35 _= 0.018, p = 0.895; LGN: F_1,35 _= 0.107, p = 0.745). While IC and LGN showed no condition-specific effects, both revealed substantially different signal behaviour. In contrast to BOLD signals at the IC (Figure 3b), BOLD signals at the LGN were consistently above zero for all conditions (Figure 3c).

## Discussion

Using individually defined anatomical ROIs and unsmoothed data, we found specific BOLD signal increases in the human SC during free visual exploration in comparison to voluntary saccades to predetermined targets. In contrast, IC and LGN revealed no comparable task-related signal changes. This finding provides evidence against unspecific (global) effects or artefacts at the dorsal brainstem contributing significantly to the observed signal changes. Moreover, signal increase in the SC was not exclusively related to differences in eye movement frequency. In contrast to previous analyses [11,25], we can now ascribe the observed signal behaviour to the SC with more certainty. Thus, it seems that the human SC is indeed involved in visual search, in accordance with observations made in non-human primates [23,24,26].

It is well known that SC neurons respond to contralateral visual stimulation without subsequent execution of motor actions [3]. It follows that additional visual stimulation in the exploration task with a high number of stimuli could have contributed to the observed signal pattern. However, while visual stimulation was comparable in the case of the exploration task and the saccade task with distracters, it was considerably different in both saccade tasks (Figure 1). Therefore, it is unlikely that significant differences between exploration on one hand and both saccade tasks on the other hand were only caused by differences of visual stimulation. Surprisingly, saccade frequency did not contribute significantly to BOLD signal variance. This could be interpreted as evidence against oculomotor functions of the superior colliculi in general. Yet, although not statistically significant, the MANOVA revealed a stronger influence of saccade frequency on SC activation compared to BOLD signals from IC and LGN. BOLD signal analysis also revealed a slight increase of activity in the SC for saccades in front of the letter field (Figure 3a, VEC). Further, analyses of BOLD signal changes using fixation as baseline might be obscured by activity of SC neurons during fixation [27]. Neurons at the rostral pole of the SC seem to inhibit the execution of (unwanted) reflexive saccades. Several studies demonstrated the necessary contribution of the SC to the execution and inhibition of reflexive saccades (for a short review cf. [28]). It was demonstrated in humans by the deterioration of reflexive saccades after disconnection of the parieto-tectal pathway [29] and the impairment of inhibitory control of reflexive saccades after lesions confined to the SC [4,6]. To conclude, our data do not provide evidence against oculomotor functions of the SC but only in favour of an increase of activity, resulting from the additional processing demands of a visual search task.

Interestingly, while IC and LGN revealed no task-related effects, signal behaviour in both structures differed substantially. While confidence intervals clearly overlapped with zero for the IC, values derived from the LGN were consistently positive in relation to the fixation baseline (Figure 3b,c). As the LGN, in contrast to the IC, should be primarily driven by visual input, this difference agrees with our assumptions and further supports the validity of our main finding. The absence of a difference between both control conditions, which differed with respect to the number of visible items in the background at the LGN, was unexpected. However, strong attentional effects have been demonstrated for the human LGN in recent studies [30]. Thus, combined with the impact of foveal magnification [30], differences between control conditions might have been obscured because both conditions required the repeated (attentional) foveation of visual targets.

Two of the few neuroimaging studies on SC functions in humans investigated the spatial topography of visual responses under binocular viewing conditions [7,8]. The authors reported a contralateral representation of visual input in the right and left SC [7,8], as well as a medio-lateral organisation of upper and lower visual field stimulation [8]. As our subjects were free to move their eyes in the exploration task and viewed the whole display with numerous fast saccades, our paradigm did not allow us to test for respective retinotopic effects of the visual input. Petit and Beauchamp [12] demonstrated comparable signal increases for eye-, head-, and gaze-movements. This result agreed with observations in monkeys [26] and supported the assumption of goal representation in the SC rather than saccade end point representation. Obviously, such a "motor map of goals", based on the selection of certain visual stimuli as relevant targets [26], constitutes an indispensable component of visual search. Recently it was also shown that saccades towards targets in the temporal hemifield were faster than saccades following nasal presentation of targets under monocular viewing conditions [31], an effect that might be driven by asymmetric visual target representations at the SC with higher activations due to temporal visual stimulation [9]. Such temporal over-representation could augment orientation towards peripheral stimuli and might play a specific role in visual search of displays with numerous stimuli in the visual periphery. However, we could not elucidate such a specific involvement of the SC using a paradigm with bilateral retinotopic stimulation under binocular viewing conditions.

Due to the use of a block design – which was chosen to optimise signal detection [32] – we can only speculate about the specific contribution of the SC to visual search. Previous studies in monkeys suggested that the selection of a behaviourally relevant target for an upcoming action (saccade or attentional shift) could be mediated by the SC [23,24]. Such a process is obviously involved in the exploration task used in our present as well as in a previous study [11,25], whereas it plays a minor role in the execution of saccades to externally determined and unequivocal target positions. Of course, other sensorimotor or cognitive operations are involved in visual searches of a complex environment as well. For example, the SC might also be involved in spatial remapping [33,34]. However, due to the constraints of the present design, these questions must remain the issue of future investigations.

## Conclusion

We demonstrated signal increases at the SC during a visual search task that could not have been generated by the visual input or oculomotor activity alone. Conducting a ROI analysis based on anatomical definitions, we demonstrated that the observed signals cannot be attributed to artefacts localised at the dorsal brainstem or to activity of nearby neural structures (e.g. pulvinar, tegmentum). Rather, they must reflect a functional contribution by the SC. Recent research in non-human primates suggests that these signal changes might be associated with the encoding of behavioural relevance and target selection during visual search. However, the specific contribution by the SC could not be elucidated with our data and therefore might comprise of other functions as well. Event-related measurements are now needed to further investigate the nature of this SC signal behaviour.

## Methods

Thirteen right-handed healthy subjects (8 m/5 f, mean age: 29 y, range: 20–47 y) participated in the experiment. All subjects gave their informed consent to participate in the study which was performed in accordance with the ethical standards established by the 1964 Declaration of Helsinki. The experimental conditions were: free visual exploration (VE), voluntary execution of saccades to constantly visible targets in the presence of a high number of distracters (VEC), and voluntary execution of saccades without further visual stimuli except for the targets (SC) (Figure 1). During exploration, the subjects were instructed to search for the target letter 'A' and to report such target letters by pressing a button with their right hand. The target was not part of the letter array in all but one block. The block with the target and thus with a button press was not included in the final data analysis.

In the saccade tasks, subjects were instructed to perform voluntary horizontal and vertical stepwise saccades alternating between different orientations (Figure 1). The whole experiment was conducted under binocular viewing conditions. Eye movements were recorded throughout the whole measurement with an infra-red eye tracker (Cambridge Research Systems) digitised at a rate of 1 kHz for offline analysis. We computed the number of saccades per condition using the first derivative of the horizontal eye movement data applying a threshold of 30 cm/s (~11.3°/s). Horizontal eye movements and oblique saccades up to ± 45° were analysed.

### Data acquisition

The experiment was conducted using a 1.5 T whole body MRI scanner (Siemens Magnetom Vision, Erlangen, Germany) using a standard head coil system. T2*-weighted echo-planar images were acquired in axial orientation (TR = 5 s, TE = 40 ms, flip angle = 90°, FOV = 192 × 192 mm, 64 × 64 matrix, 44 slices, slice thickness 3 mm) for blood oxygen level-dependent (BOLD) based imaging. The planes were individually oriented parallel to the AC-PC line and covered the whole cerebral volume including the superior half of the cerebellum in all subjects. Additionally, high-resolution T1-weighted anatomical volumes were acquired using a MP-RAGE sequence (TR = 9.7 ms, TE = 4 ms, FOV = 256 × 256 mm, 256 × 256 matrix, 128 sagittal slices, slice thickness 1.5 mm).

### Image analysis

Image analysis was carried out using Statistical Parametric Mapping (SPM2, Wellcome Department of Imaging Neuroscience, London, UK) implemented in MatLab 7 (Mathworks, Inc., Sherborn, MA, USA). The first four images of each measurement were discarded to allow the MRI signal to reach a steady state. The remaining images of each subject were realigned to the first image to correct for head movements during the experiment. Following spatial image realignment, we unwarped images to correct for EPI distortions due to motion. Subsequently, the anatomical T1 volume was co-registered to the mean of the functional EPI images and aligned to the SPM T1 template. The calculated non-linear transformation was applied to all functional images for spatial normalisation, resampling images at a resolution of 3 × 3 × 3 mm^3^. Images were not smoothed to optimise localisation of signal changes. The statistical analysis of the imaging data included the removal of low-frequency drifts in the signal using a high-pass filter of 300 s. Temporal autocorrelation of errors was accommodated by an AR(1) model implemented in SPM2. The different experimental conditions were modelled by a boxcar function convolved with a haemodynamic response function as implemented in SPM2. We defined a design matrix comprising of four parameters for the conditions of interest. Additionally, six covariates to capture residual movement-related artefacts (three rigid-body translations and three rotations determined from the realignment procedure) were included.

We determined individual regions of interest (ROI) comprising of the SC, IC, and LGN for each subject based on the individual normalised T1-weighted image using MRIcro [35] (Figure 2). We used the MarsBar toolbox for SPM2 [36,37]) to extract time courses from voxels within the respective ROI and to calculate the median time course across these voxels in each subject. MarsBar calculates percent signal change associated with an experimental condition (e.g. visual search) as the maximum of the time course of the estimated event for this condition, divided by the mean signal across the time course of the whole session, and multiplied by 100. The average signal used in this calculation is based on all conditions and is identified as the beta value for the mean column of the SPM regression analysis. Subsequently, a multivariate analysis of variance (MANOVA) on the percent signal changes in SC, IC, and LGN including the mean frequency of saccades per condition as covariate was conducted using SPSS 14.

## Authors' contributions

MH conceived the experiment, conducted measurements, analysed data, and drafted the manuscript. ME operated the MRI scanner and assisted in data analysis. HOK participated in the task design and contributed to the preparation of the manuscript. All authors read and approved the final manuscript.

## References

[B1] Moschovakis AK (1996). The superior colliculus and eye movement control. Curr Opin Neurobiol.

[B2] Sparks DL (2002). The brainstem control of saccadic eye movements. Nat Rev Neurosci.

[B3] May PJ (2005). The mammalian superior colliculus: Laminar structure and connections. Prog Brain Res.

[B4] Pierrot-Deseilligny C, Rosa A, Masmoudi K, Rivaud S, Gaymard B (1991). Saccade deficits after a unilateral lesion affecting the superior colliculus. J Neurol Neurosurg Psychiatry.

[B5] Sapir A, Soroker N, Berger A, Henik A (1999). Inhibition of return in spatial attention: direct evidence for collicular generation. Nat Neurosci.

[B6] Sereno AB, Briand KA, Amador SC, Szapiel SV (2006). Disruption of reflexive attention and eye movements in an individual with a collicular lesion. Journal of Clinical and Experimental Psychology.

[B7] DuBois RM, Cohen MS (2000). Spatiotopic organization in human superior colliculus observed with fMRI. Neuroimage.

[B8] Schneider KA, Kastner S (2005). Visual Responses of the Human Superior Colliculus: A High-Resolution Functional Magnetic Resonance Imaging Study. J Neurophysiol.

[B9] Sylvester R, Josephs O, Driver J, Rees G (2007). Visual fMRI Responses in Human Superior Colliculus Show a Temporal-Nasal Asymmetry That Is Absent in Lateral Geniculate and Visual Cortex. J Neurophysiol.

[B10] Calvert GA, Hansen PC, Iversen SD, Brammer MJ (2001). Detection of audio-visual integration sites in humans by application of electrophysiological criteria to the BOLD effect. Neuroimage.

[B11] Gitelman DR, Parrish TB, Friston KJ, Mesulam MM (2002). Functional anatomy of visual search: regional segregations within the frontal eye fields and effective connectivity of the superior colliculus. Neuroimage.

[B12] Petit L, Beauchamp MS (2003). Neural Basis of Visually Guided Head Movements Studied With fMRI. J Neurophysiol.

[B13] Enzmann DR, Pelc NJ (1992). Brain motion: measurement with phase-contrast MR imaging. Radiology.

[B14] Poncelet BP, Wedeen VJ, Weisskoff RM, Cohen MS (1992). Brain parenchyma motion: measurement with cine echo-planar MR imaging. Radiology.

[B15] Müri R (2005). MRI and fMRI analysis of oculomotor function. Prog Brain Res.

[B16] Freedman EG, Stanford TR, Sparks DL (1996). Combined eye-head gaze shifts produced by electrical stimulation of the superior colliculus in rhesus monkeys. J Neurophysiol.

[B17] Freedman EG, Sparks DL (1997). Eye-head coordination during head-unrestrained gaze shifts in rhesus monkeys. J Neurophysiol.

[B18] Bergeron A, Guitton D (2000). Fixation neurons in the superior colliculus encode distance between current and desired gaze positions. Nat Neurosci.

[B19] Bergeron A, Guitton D (2002). In multiple-step gaze shifts: Omnipause (OPNs) and collicular fixation neurons encode gaze position error; OPNs gate saccades. J Neurophysiol.

[B20] Bergeron A, Matsuo S, Guitton D (2003). Superior colliculus encodes distance to target, not saccade amplitude, in multi-step gaze shifts. Nat Neurosci.

[B21] Basso MA, Wurtz RH (1997). Modulation of neuronal activity by target uncertainty. Nature.

[B22] Basso MA, Wurtz RH (1998). Modulation of neuronal activity in superior colliculus by changes in target probability. J Neurosci.

[B23] McPeek RM, Keller EL (2002). Saccade Target Selection in the Superior Colliculus During a Visual Search Task. J Neurophysiol.

[B24] McPeek RM, Keller EL (2002). Superior Colliculus Activity Related to Concurrent Processing of Saccade Goals in a Visual Search Task. J Neurophysiol.

[B25] Himmelbach M, Erb M, Karnath HO (2006). Exploring the visual world: The neural substrate of spatial orienting. Neuroimage.

[B26] Krauzlis RJ, Liston D, Carello CD (2004). Target selection and the superior colliculus: Goals, choices and hypotheses. Vision Res.

[B27] Munoz DP, Wurtz RH (1993). Fixation cells in monkey superior colliculus. I. Characteristics of cell discharge. J Neurophysiol.

[B28] Leigh RJ, Kennard C (2004). Using saccades as a research tool in the clinical neurosciences. Brain.

[B29] Gaymard B, Lynch J, Ploner C, Condy C, Rivaud P (2003). The parieto-collicular pathway: Anatomical location and contribution to saccade generation. Eur J Neurosci.

[B30] Kastner S, Schneider KA, Wunderlich K (2006). Beyond a relay nucleus: neuroimaging views on the human LGN. Prog Brain Res.

[B31] Kristjansson A, Vandenbroucke MWG, Driver J (2004). When pros become cons for anti-versus prosaccades: factors with opposite or common effects on different saccade types. Exp Brain Res.

[B32] Birn R, Cox R, Bandettini P (2002). Detection versus estimation in event-related fMRI: choosing the optimal stimulus timing. Neuroimage.

[B33] Walker MF, Fitzgibbon EJ, Goldberg ME (1995). Neurons in the monkey superior colliculus predict the visual result of impending saccadic eye movements. J Neurophysiol.

[B34] Wurtz RH, Sommer MA (2004). Identifying corollary discharges for movement in the primate brain. Prog Brain Res.

[B35] Rorden C, Brett M (2000). Stereotaxic display of brain lesions. Behav Neurol.

[B36] Brett M, Anton J-L, Valabregue R, Poline J-B (2002). Region of interest analysis using an SPM toolbox [abstract]. Neuroimage.

[B37] MarsBaR region of interest toolbox for SPM. http://marsbar.sourceforge.net/.

